# All-trans retinoic acid and protein kinase C α/β1 inhibitor combined treatment targets cancer stem cells and impairs breast tumor progression

**DOI:** 10.1038/s41598-021-85344-w

**Published:** 2021-03-15

**Authors:** Damian Emilio Berardi, Lizeth Ariza Bareño, Natalia Amigo, Luciana Cañonero, Maria de las Nieves Pelagatti, Andrea Nora Motter, María Agustina Taruselli, María Inés Díaz Bessone, Stefano Martin Cirigliano, Alexis Edelstein, María Giselle Peters, Miriam Diament, Alejandro Jorge Urtreger, Laura Beatriz Todaro

**Affiliations:** 1 Research Area, Instituto de Oncología “Ángel H. Roffo”, Área Investigación, Universidad de Buenos Aires, Av. San Martín 5481, C1417DTB Buenos Aires, Argentina; 2 Facultad de Ciencias Exactas y Naturales, Departamento Química Biológica, Instituto de Química Biológica de la Facultad de Ciencias Exactas Y Naturales (IQUIBICEN), Universidad de Buenos Aires, CONICET-Universidad de Buenos Aires, Buenos Aires, Argentina; 3 Unidad Operativa Centro de Contención Biológica de la Administracion Nacional de Laboratorios e Institutos de Salud (UOCCB-ANLIS), “Dr. Carlos G. Malbrán”, Buenos Aires, Argentina; 4 Member of the Scientific Research Career of the Consejo Nacional de Investigaciones Científicas y Técnicas (CONICET), Buenos Aires, Argentina; 5 Present Address: The Ben May Department for Cancer Research, The Gordon Center for Integrative Sciences, The University of Chicago, Chicago, IL USA; 6 Present Address: Meyer Cancer Center, Weill Cornell Medicine, New York, NY USA; 7 Present Address: Instituto de Nanosistemas, Universidad Nacional de San Martín, Campus Miguelete, San Martín, Argentina

**Keywords:** Cell biology, Oncology

## Abstract

Breast cancer is the leading cause of cancer death among women worldwide. Blocking a single signaling pathway is often an ineffective therapy, especially in the case of aggressive or drug-resistant tumors. Since we have previously described the mechanism involved in the crosstalk between Retinoic Acid system and protein kinase C (PKC) pathway, the rationale of our study was to evaluate the effect of combining all-trans-retinoic acid (ATRA) with a classical PCK inhibitor (Gö6976) in preclinical settings. Employing hormone-independent mammary cancer models, Gö6976 and ATRA combined treatment induced a synergistic reduction in proliferative potential that correlated with an increased apoptosis and RARs modulation towards an anti-oncogenic profile. Combined treatment also impairs growth, self-renewal and clonogenicity potential of cancer stem cells and reduced tumor growth, metastatic spread and cancer stem cells frequency in vivo. An in-silico analysis of “Kaplan–Meier plotter” database indicated that low PKCα together with high RARα mRNA expression is a favorable prognosis factor for hormone-independent breast cancer patients. Here we demonstrate that a classical PKC inhibitor potentiates ATRA antitumor effects also targeting cancer stem cells growth, self-renewal and frequency.

## Introduction

Breast cancer is one of the most common malignancy and the leading cause of cancer death among women worldwide^1^. Breast cancer represents a group of tumors with different biologic behavior and high clinical variability. Although, therapies before and/or after surgery (neoadjuvant or adjuvant) improve prognosis, recurrence and resistance to traditional therapies are challenges to solve. In this sense, triple negative breast cancer subtype (lacking estrogen expression, progesterone and HER2 receptors), is characterized by a short-term response to chemo and radio therapies, but after patients´ relapse, no therapeutic alternatives remain available. The main responsible of this resistance is the presence of cancer stem cells (CSC), which usually present different biological behavior and growth kinetics than the rest of tumor. A large body of evidence suggests that CSCs self-renewal ability allows them to differentiate into heterogeneous lineages of cancer cells in response to chemotherapeutic agents, favoring resistance and inducing relapse^2^. Moreover, due to CSCs plasticity, this tumor component has been considered as the metastasis seed^3,4^. Indeed, it has been proposed the existence of latent CSC in the bone marrow, and when these cells become active, they cause recurrence even many years later^5^. For these reasons, it is imperative to study and develop new therapies targeting both bulk tumor and cancer stem cells in order to prevent tumor growth and metastatic spread mainly in hormone-independent breast cancer patients, which lack effective second-line therapeutic alternatives.

A group of molecules derived from vitamin A (the retinoids) have been successfully used for acute promyelocytic leukemia treatment^6^. Although this differentiation therapy^7,8^ has controversial results in solid-tumors treatment, some phase II clinical trials are being evaluated nowadays^9,10^. Retinoids are ligands of retinoic acid receptors (RARs) α, β and γ, which are strong cell differentiation agents for both epithelial and non-epithelial cells, leading growth arrest^11^. It has been reported that all-trans-retinoic acid (ATRA) can induce differentiation of breast CSCs^12^ sensitizing them to chemotherapeutic agents^13^, indicating the importance that this compound could display in cancer clinical settings.

In recent years interest of combining ATRA with other drugs for cancer treatment has emerged. In this sense, we have previously described the mechanism involved in ATRA and protein kinase C (PKC) crosstalk that would be associated with malignant phenotype reversion^14^. Nevertheless, there are almost no reports showing the effect of combining retinoids with signal transduction pathways inhibitors in the bibliography, till nowadays^15^.

PKC is a family of lipid-dependent serine/threonine kinases that play central roles in signal transduction pathways controlling proliferation, differentiation, apoptosis, and malignant transformation^16^. PKC isoforms have been grouped into three families: classical (α, βI, βII, and γ), novel (δ, ε, η and θ), and atypical (ζ and λ/ι), based on their structural similarities and biochemical properties^16^. PKCα has been considered as an aggressiveness marker for breast cancer^17^ and also a key component in CSCs signaling^18^. In addition, an increase in classical PKC activity and/or expression has been reported for a large variety of ATRA treated cells, such as breast^19^, pancreatic^20^, melanoma^21^, and neuroblastoma cells^22^.

Considering, the importance of a combined therapy in order to target all tumor cell components by altering multiple signaling pathways, here we proposed to evaluate potential application of ATRA together with Gö6976, a classical PKC inhibitor, on malignant progression. Although drug combination is a common practice in oncology, in order to reduce adverse effects, the combination proposed has not been evaluated previously. We employed human SKBR3 and HCC38 breast cancer cell lines and a BALB/c syngeneic mammary model^23,24^ (LM38-LP) both lacking hormone receptors, in order to resemble patients’ clinical conditions.

Here we reported that ATRA and Gö6976 combined treatment target cancer stem cells population and impair breast tumor progression, both in vitro and in vivo, in a synergist manner. Altogether results presented in this manuscript encourage the design of novel treatments employing retinoids together with PKC inhibitors.

## Materials

### Studies in vitro

#### Reagents and antibodies

Medium for cell culture and agarose were obtained from Life Technologies Inc. (Rockville, MD). Fetal bovine serum (FBS) was from GEN (Buenos Aires, Argentina). Acrylamide and retinoids were from Sigma (St. Louis, MO). All other reagents for polyacrylamide gel electrophoresis were obtained from Bio-Rad (Richmond, CA). Monoclonal antibodies anti Actin were purchased from Santa Cruz Biotechnology (Santa Cruz, CA). Monoclonal antibodies for PKCα and PKCβ detection were purchased from Santa Cruz Biotechnology (Santa Cruz, CA) and LC3-I/II and SQSTM1/p62 antibodies were from Cell Signaling Technology (Danvers, MA). Horseradish peroxidase conjugated anti-rabbit or anti-mouse antibodies were obtained from Sigma (St. Louis). Hybond-P membranes for blotting and chemiluminescence reagents (ECL) were from Amersham (Aylesbury, UK). Classical PKC inhibitor (Gö6976) was obtained from Calbiochem (Billerica, MA). Annexin V Kit was purchased from Thermo Fisher Scientific (Waltham, MA).

#### Cell lines and culture conditions

LM38-LP, SKBR3 and HCC38 cell lines were used in this study. LM38-LP cell line was previously established in our laboratory from spontaneous BALB/c mammary papillary adenocarcinoma with tumorigenic and metastatic capacities^23^. This cell line is composed of subpopulations antigenically characterized as luminal epithelial (LEP) and myoepithelial (MEP), and bipotent cancer stem cells (CSC) that were able to differentiate to both LEP and MEP cells^24^. LM38-LP and SKBR3 cells were grown in DMEM/F12 medium with non-essential amino acids and 2 μM L-glutamine (Gibco, Life Technologies, Rockville, MD), and HCC38 cells were growth in DMEM (Gibco, Life Technologies). In all cases, cells were supplemented with 10% FBS and cultured at 37 °C in plastic tissue culture flasks (Greiner Bio-One, Frickenhausen, Germany) in a humidified 5% CO_2_/air atmosphere. Serial passages were carried out through treatment of sub-confluent monolayers with 0.25% trypsin and 0.02% EDTA in Ca^2+^ and Mg^2+^-free PBS (Gibco, Carlsbad, CA).

#### Proliferation assays

Proliferative capacity was determined by assessing LM38-LP, SKBR3 and HCC38 cell number during exponential growth phase of cell monolayers. Briefly: 4 × 10^5^ cells were seeded onto 35 mm Petri dishes and treated with ATRA (0.25–1 μM) and/or Gö6976 (0.25–1 µM) once a day during 96 h (n = 3) in culture media supplemented with 80 µg/ml gentamicin and 10% FBS. At different times after seeding, cells were washed with PBS, detached with 0.05% trypsin, and counted using a hemocytometer and trypan blue exclusion.

#### Analysis of drug interactions

Drug interaction results from cell proliferation assay were examined by the method of Chou and Talalay^25,26^ using commercially available software CalcuSyn^27^ (Biosoft, Ferguson, MO). Combination index (CI) is a quantitative measurement of the degree of interaction between two or more drugs: CI < 0.7 indicates synergism between the drugs, CI > 0.7 and CI < 1 indicates additivity and CI > 1 denotes antagonism.

#### Mammosphere assay

To enrich cancer stem/progenitor cell component, a suspension containing 1 × 10^4^ LM38-LP and HCC38 cells were seeded onto low attachment 35 mm culture dishes in serum‐free DMEM‐F12 medium supplemented with B27 without vitamin A (Life Technologies, Rockville, MD) plus 20 ng/ml epidermal growth factor (BD Biosciences, San Diego, CA). When mammosphere were formed (10 cells approx.), they receive the following treatments: ATRA (0.5 μM), Gö6976 (0.5 μM), their combination or vehicle alone for 96 h. Alternatively, LM38-LP mammospheres were treated with LE 135 (RAR β antagonist, 200 nM) or MM 11253 (RARγ antagonist, 1 µM) combined or not with ATRA (0.5 μM). Photographs were taken under contrast microscopy.

#### Mammosphere size determination

After 96 h treatment, mammospheres were observed under an inverted microscope (Nikon Eclipse TE 2000-S) and 10 random fields were digitally photographed using a digital camera. Mammosphere diameters were measured on the long axis using Image J software and average size was calculated.

#### Secondary mammosphere assay

Primary mammospheres were dissociated using 0.05% trypsin for 15 min at 37 °C in order to obtain a single‐cell suspension. Then, cell suspension was seeded and cultured as described above for mammosphere assay, in order to obtain a new generation of mammospheres. Sphere formation was assessed after 5 days and mammospheres size and number was recorded.

#### Clonogenic assay

After 96 h treatment, primary mammospheres were enzymatically dissociated as described above and a suspension containing 1 × 10^3^ cells was plated in adherent conditions. After 7 days culture, colonies were fixed with methanol:acetic (3:1) and stained with crystal violet.

#### Analysis of cell cycle distribution by flow cytometry

Monolayers, treated for 96 h with ATRA (0.5 µM), Gö6976 (0.5 µM), ATRA plus Gö6976 or vehicle (as control) were detached with trypsin (0.05%), washed with PBS and fixed with ethanol (70%). Cells were stained with propidium iodide (100 µg/ml) and DNA content was examined by flow cytometry employing a Coulter EPICS Elite ESP cytometer (Beckman coulter, Fullerton, CA).

##### Detection of apoptosis by annexin V assay

Cell monolayers were treated with ATRA (0.5 µM), Gö6976 (0.5 µM), their combination or vehicle alone for 48 h. Then, cells were collected, and apoptotic cells were quantified as described by the manufacturer. Briefly, cells (1 × 10^6^) were washed and resuspended in 100 μl 1X binding buffer. Then, cells were incubated 15 min in darkness at room temperature with 5 μl of Alexa488-conjugated Annexin V. Cells were washed with 1X binding buffer and finally 5 μl of propidium iodide was added. Cells were mixed in darkness at room temperature for 10 min, then 300 μl of 1X binding buffer was added, and cells were mixed in an ice bath at dark. Cell suspension was examined under 488 nm excitation wavelength by flow cytometry using an Epics Elite ESP coulter cytometer (Beckman coulter, Fullerton, CA).

##### Western blot

Western blot (WB) assays were performed as previously described by Berardi et al.^14^, employing cell lysates prepared from monolayers treated for 48 h with ATRA (0.5 µM), Gö6976 (0.5 µM), their combination or vehicle alone as control.

##### RT-qPCR

Subconfluent cultures of each cell line were treated for 48 h with ATRA (0.5 µM), Gö6976 (0.5 µM), their combination or vehicle alone. Total RNA was prepared using Tri Reagent (Merck, Darmstadt, Germany). cDNA was prepared with the iScript cDNA synthesis kit (Bio Rad) and amplified by real-time PCR using a CFX96 Real-Time PCR detection systems kit (Bio-Rad) and SYBR green PCR master mix (Applied Biosystems, Carlsbad, CA). PCR products were obtained using primers indicated in Table S1. GAPDH was used as housekeeping gene. Relative changes in gene expression were calculated with the 2-∆∆CT or 2-∆RAR∆CT method^28^.

##### Wound migration assay

Subconfluent LM38-LP and SKBR3 monolayers were treated with Gö6976 (0.5 µM) and/or ATRA (0.5 µM) or vehicle as control for 48 h. Then, wounds of approximately 400 μm width were performed and cells were allowed to migrate to the cell-free area for a period of 12 h for LM38-LP or 24 h for SKBR3 cells. The same spot was photographed at different times and migratory area was quantified with ImageJ software. Cell migration was expressed as percentage of area occupied by the migratory cells in the original cell-free wounded area. Cell viability was not affected by ATRA or Gö6976 doses used in this experiment.

##### Preparation of conditioned media (CM)

Metalloproteinases (MMPs) activity was evaluated in CM after the treatment with ATRA and/or Gö6976. Briefly: cell monolayers growing in 6 well plates were treated with ATRA and/or Gö6976 during 48 h. Then monolayers were extensively washed with PBS to eliminate serum traces and 500 µl of serum-free medium was added and the incubation continued for 20 h. Finally, CM were harvested, remaining monolayers were lysed with 1% Triton X-100 in PBS and cell protein content was determined. CM samples were aliquoted, stored at -20 °C and used once after thawing.

##### Determination MMPs secreted activity by zymography

Briefly: samples were run on a 9% SDS-PAGE gels co-polymerized with gelatin (1 mg/ml). After running, gels were washed in 2% Triton X-100 and incubated at 37 °C for 48 h in a buffer containing 0.25 M Tris–HCl/1 M NaCl/25 mM CaCl_2_ buffer (pH 7.4) for activity detection. Finally, gels were stained with Coomassie Blue (0.1% Coomassie R-250, 10% acetic acid and 30% methanol). Activity bands were visualized as negative staining and were quantified using the Gel Pro Analyzer program. Data was expressed in arbitrary units and was relativized to the protein content of cell lysates.

### Studies in vivo

#### Animals

For the in vivo experimental procedures, we employed randomized inbred female BALB/c mice of 2–4 months old (20–25 g) that were obtained from the Animal Care Division of the Institute of Oncology “A. H. Roffo”^14,24^. Mice were under automatic 12 h light/12 h darkness schedule, kept 5 per cage and provided with tap water *ad libitum* and sterile pellets (Cooperacion, SENASA N° 04–288/A). Rectangular polycarbonate cages (20 × 29 × 14 cm) with irradiated pinewood (15 KGy) were employed for housing. All animal studies were conducted in accordance with the standards of animal care as outlined in the NIH and ARRIVE Guidelines for the Care and Use of Laboratory Animals. Besides, the Committee for the Use and Care of laboratory Animals (CICUAL) of the Institute of Oncology “A. H. Roffo” (University of Buenos Aires) had approved our protocols.

We established humane endpoint when mice met one of the following signs: Bristling coat and/or hemorrhagic diarrhea, loss of > 20% of the initial weight or lethargy. Animals were euthanized by CO_2_ inhalation.

#### Orthotopic tumor growth and spontaneous metastatic ability

Tumor growth and spontaneous metastatic ability were evaluated as previously described in detail^14,24^. In brief, mice were inoculated orthotopically into the fat pad of the 4^th^ mammary gland with 2 × 10^5^ LM38-LP cells (n = 5 for group, 20 animals in each experiment). Five days after cell inoculation, mice were anesthetized injecting a combination of ketamine (100 mg/kg) and xylazine (5 mg/kg) intraperitoneally. Then each mouse received a subcutaneous silastic pellet containing ATRA (10 mg) or an empty pellet as control. Gö6976 (60 mg/kg) was locally administered twice a week. The control group received the same volume of solvent (0.2 ml of 0.1% DMSO physiologic solution). Mice were monitored daily. Twice a week, tumor diameters were measured with a sliding caliper and tumor volume was calculated using the following formula: Dxd^2^/2, where D is the longest and d is the shortest diameter. Twenty-one days after tumor treatment, mice were sacrificed as described above and necropsied. Lungs were removed and fixed in Bouin’s solution to investigate the presence of spontaneous metastases. The number of surface lung nodules was recorded. Liver, kidney, and spleen were also fixed and examined for the presence of metastatic nodules.

#### LM38-LP tumor cell suspension preparation and limiting dilution assay

LM38-LP tumors harvested post-treatment were minced and digested in digestion media as previously described in detail^29^. Next, LM38-LP tumor-derived were plated at 10 and 100 cells per well into a ultra-low attachment 96 well plate. 5 days after plating, mammospheres number found in each well was quantified under microscope. Cancer stem cell frequency and p-values were calculated by using ELDA software^30^.

### Statistical analysis

All assays were performed in triplicate, and independent experiments repeated at least twice. Statistical differences between groups were calculated by applying ANOVA, Student’s t or Kruskal–Wallis tests, as indicated. A *p* value < 0.05 was considered statistically significant.

### Studies in silico

#### Bioinformatic analysis from Kaplan Meier Plot database of PRKCA and RARA expression

A Kaplan–Meier survival database that contains survival information of 801 estrogen receptor negative breast cancer patients and gene expression data at diagnosis obtained by using Affymetrix microarrays^31^ was employed. Probes set were 213093_at (PRKCA) and 203750_at (RARA) and split patients by auto select best cut off into a low-expression group and a high-expression group. Relapse free survival (RFS) curves were plotted according to the Kaplan Meier method and evaluated by the log-rank test.

### Ethics approval and consent to participate

All animal studies were conducted in accordance with the standards of animal care as outlined in the NIH and ARRIVE Guidelines for the Care and Use of Laboratory Animals. Protocols have the approval of the Committee for the Use and Care of laboratory Animals (CICUAL) of the Institute of Oncology “A. H. Roffo”, University of Buenos Aires.

## Results

### ATRA and Gö6976 treatments reduce breast cancer and breast cancer stem cells proliferation

**Figure 1:**
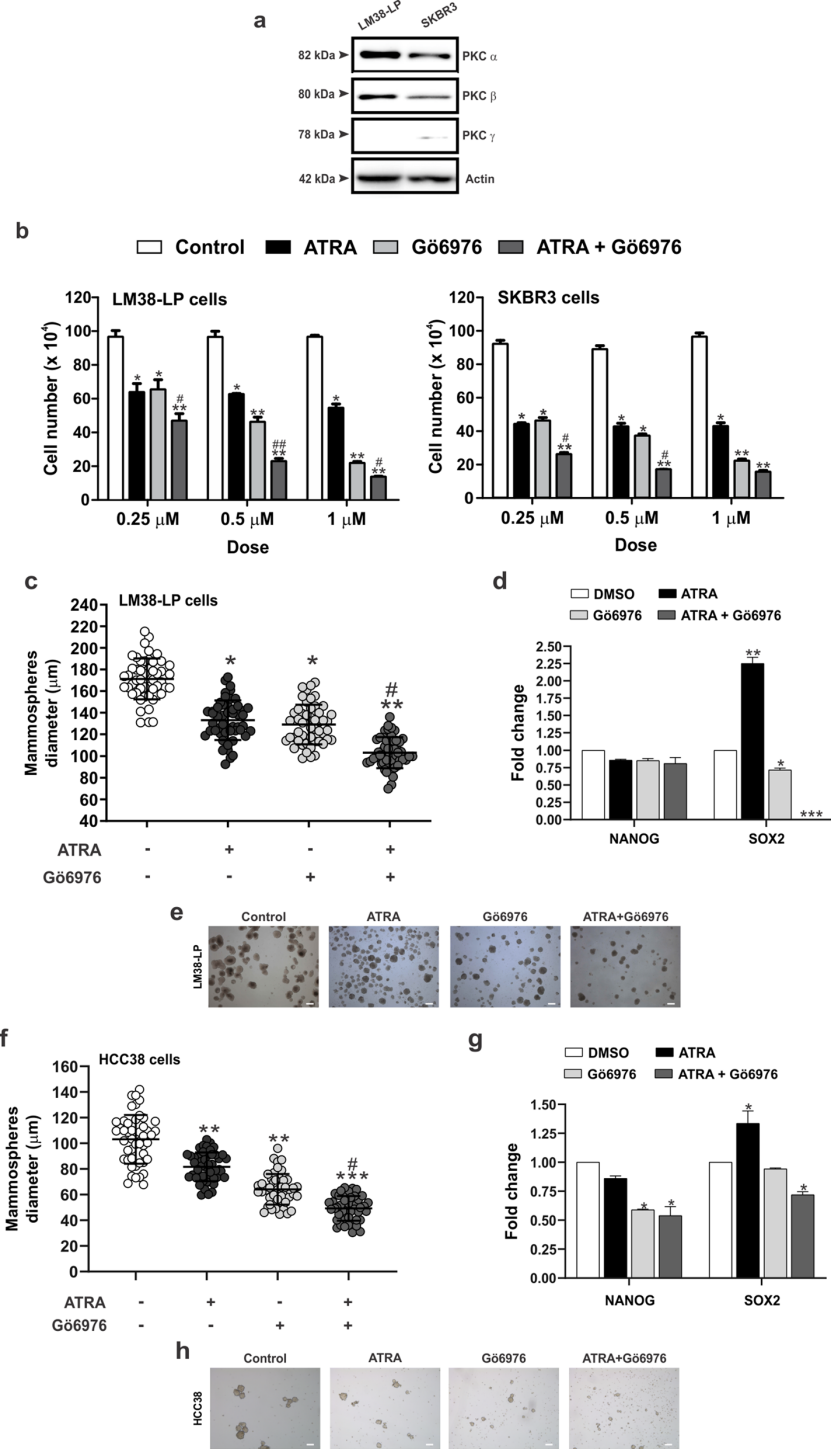
(**a**) Expression of classical PKC isoforms. Whole cell lysates prepared from LM38-LP and SKBR3 cell lines were resolved on sodium dodecyl sulfate–polyacrylamide gel electrophoresis and blotted with antibodies against PKC α, β and γ. Actin expression levels was used as protein loading control. (**b**) LM38-LP and SKBR3 cell number was assessed 96 h after treatments with ATRA (0.25–1 µM) and/or Gö6976 (0.25–1 µM) or vehicle as control. (**c**) LM38-LP mammospheres diameter was measured 96 h after treatments with ATRA (0.5 µM) and/or Gö6976 (0.5 µM) or vehicle as control. (**d**) LM38-LP cells were treated with ATRA (0.5 µM) and/or Gö6976 (0.5 µM) or vehicle as control for 48 h and then RNA was isolated. Nanog and Sox2 expression was analyzed by RT-qPCR. The fold of change of mRNA levels was calculated using the ΔΔCt method with GAPDH used as an internal control. Histograms represent mean ± S.D. (**e**) Representative photographs of LM38-LP mammospheres after 96 h treatments. (**f**) HCC38 mammospheres diameter was measured 96 h after treatments with ATRA (0.5 µM) and/or Gö6976 (0.5 µM) or vehicle as control. (**g**) HCC38 cells were treated with ATRA (0.5 µM) and/or Gö6976 (0.5 µM) or vehicle as control for 48 h and then RNA was isolated. Nanog and Sox2 expression was analyzed by RT-qPCR. The fold of change of mRNA levels was calculated using the ΔΔCt method with GAPDH used as an internal control. Histograms represent mean ± S.D (**h**) Representative photographs of HCC38 mammospheres after 96 h treatments. Scale bar 100 µm. Data represent the mean ± S.D. *p < 0.05 versus control, **p < 0.01 versus control, ***p < 0.001 versus control, ^#^*p* < 0.05 versus Gö6976, ^##^*p* < 0.01 versus Gö6976 (ANOVA test). Three independent experiments were performed.

First, we have analyzed the expression of PKC α, β and γ by WB in LM38-LP and SKBR3 cell lines, since this classical PKC isoforms are the main target of Gö6976. As shown in Fig. 1a, PKCα showed high expression levels in both cell lines, while PKCβ showed moderated expression. PKCγ was almost undetectable, as previously reported for mammary tissues^32^.

LM38-LP and SKBR3 cells were treated with ATRA or Gö6976 at a range of doses from 0.25 to 1 μM for both drugs. Additionally, ATRA and Gö6976 combination employing those doses was also tested. After 4 days treatment, the effects of single agents or drug combinations on LM38-LP and SKBR3 cell number was examined. As shown in Fig. 1b, we could determine that ATRA and Gö6976 treatment alone led to a significant decrease in the proliferative potential of both cell lines. Moreover, ATRA and Gö6976 combination also reduced proliferative capacity but in a higher degree than each treatment alone. The stronger effect was achieved employing 0.5 μM of both compounds thus, this concentration was chosen for the next set of experiments.

In order to evaluate the effect of ATRA and Gö6976 on CSC proliferation, a mammosphere assay was performed. The stem/progenitor component characteristics of LM38-LP cell line was described elsewhere^24^. SKBR3 cells were not employed in these assays since they are not able to form mammospheres^33^, thus we used HCC38 human breast cancer cell line, where only combined ATRA and Gö6976 treatment was able to inhibit the proliferative capacity (Fig. S1). We observed that ATRA or Gö6976 treatment alone slightly reduced LM38-LP and HCC38 CSC proliferation between 20 and 30% (Fig. 1c,e,f,h). Moreover, ATRA and Gö6976 combination highly reduced proliferative capacity of LM38-LP and HCC38 CSC between 50 and 60% (Fig. 1c,e,f,h). Surprisingly, when we analyzed CSC markers by qPCR after 48 h of treatments, we observed that ATRA increase SOX2 expression in both cell lines (Fig. 1d,g). On the other hand, Gö6976 treatment led to reduce only NANOG or SOX2 depending the cell line (Fig. 1d,g). Remarkably, only combined treatment led to a significant reduce of both CSC markers in both cell lines.

### ATRA and Gö6976 combination have a synergic interaction

Next, we analyzed whether the growth inhibition observed under combined treatment was due to a synergistic interaction between the drugs employed. Results obtained in proliferation assays were expressed as the percentage of control and drug interactions was analyzed by Chou-Talalay’s method^25,26^. As shown in Table 1, each single agent displayed a dose-dependent inhibition of cell proliferation. All ATRA and Gö6976 combinations studied exhibited a synergistic effect shown by their Combination index (CI) lower than 0.7.

**Table 1:** Proliferation assay, determination of the combination Index (CI).

Cell fraction affected at 96 h				
**Dose**	**ATRA**	**Gö6976**	**ATRA + Gö6976**	**CI**
**LM38-LP**				
0.25 μM	0.34 ± 0.02	0.32 ± 0.02	0.52 ± 0.02	0.62 ± 0.02
0.5 μM	0.35 ± 0.01	0.52 ± 0.01	0.76 ± 0.02	0.52 ± 0.01
1 μM	0.44 ± 0.01	0.78 ± 0.02	0.86 ± 0.04	0.66 ± 0.03
**SKBR3**				
0.25 μM	0.52 ± 0.03	0.50 ± 0.02	0.72 ± 0.06	0.45 ± 0.05
0.5 μM	0.52 ± 0.02	0.58 ± 0.02	0.81 ± 0.03	0.31 ± 0.02
1 μM	0.56 ± 0.02	0.79 ± 0.01	0.85 ± 0.01	0.69 ± 0.01

LM38-LP and SKBR3 cells number was assessed 96 h after treatments with ATRA (0.25–1 µM) and/or Gö6976 (0.25–1 µM). Each data point represents the mean ± standard error of three independent experiments. CI < 0.7 indicates synergism, CI > 0.7 and CI < 1 indicates additivity, and CI > 1 denotes antagonism.

### ATRA and Gö6976 combination impair cancer stem cell self-renewal and clonogenicity

**Figure 2:**
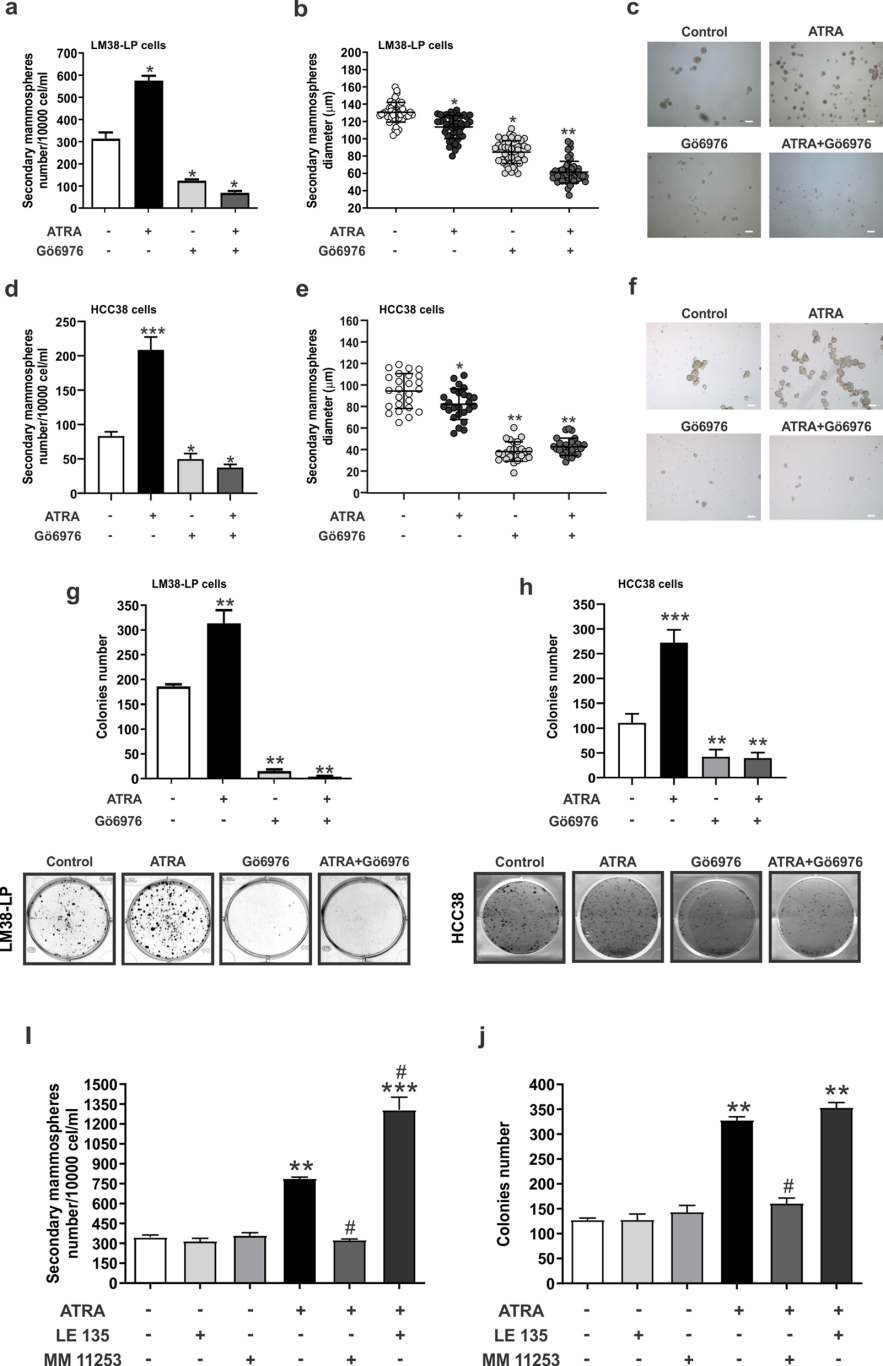
Modulation of self-renewal and clonogenic capacity of LM38-LP mammospheres. (**a**) Determination of LM38-LP secondary mammosphere number after 5 days in culture. (**b**) Determination of LM38-LP secondary mammosphere diameter after 5 days in culture. (**c**) Representative photographs of LM38-LP secondary mammospheres derived from pre-treated primary mammospheres are shown. (**d**) Determination of HCC38 secondary mammosphere number after 5 days in culture. (**e**) Determination of HCC38 secondary mammosphere diameter after 5 days in culture. (**f**) Representative photographs of HCC38 secondary mammospheres derived from pre-treated primary mammospheres are shown. Scale bar 100 µm. (**g**) Clonogenic capacity of LM38-LP cells derived from pre-treated mammospheres. Inset: Representative photographs of LM38-LP colonies derived from pre-treated mammospheres. (**h**) Clonogenic capacity of HCC38 cells derived from pre-treated mammospheres Inset: Representative photographs of HCC38 colonies derived from pre-treated mammospheres. (**i**) Determination of LM38-LP secondary mammosphere number derived from primary mammospheres treated with ATRA (0.5 µM) and/or MM 11,253 (1 µM), LE 135 (200 nM) or vehicle as control. (**j**) Clonogenic capacity of LM38-LP cells derived from pre-treated mammospheres. Data represent the mean ± S.D., *p < 0.05 versus control, **p < 0.01 versus control, ***p < 0.001 versus control, ^#^*p* < 0.05 versus ATRA (ANOVA test).

In order to determine if the different treatments affected cancer stem cell self-renewal and clonogenicity, LM38-LP and HCC38 mammospheres were pre-treated for 96 h with ATRA and/or Gö6976 and a secondary mammosphere assay was performed. Although ATRA treatment increased the number of secondary mammospheres (Fig. 2a,c,d,f) and the clonogenic capacity (Fig. 2g,h), mammospheres diameters were smaller (Fig. 2b,e). On the other hand, Gö6976 pre-treatment was able to reduce both the number and diameter of secondary mammospheres (Fig. 2a–f), as well as clonogenicity (Fig. 2g,h). Combined treatment impairs the increase of cancer stem cells self-renewal and clonogenicity induced by ATRA and led to a significantly diameter reduction of secondary mammospheres (Fig. 2a–h).

Since, retinoic acid drive many of its biology effects through RARs activation, we analyze which RARs isotype would be involved in cancer stem self-renewal and clonogenicity upon ATRA treatment. The RARγ antagonist (MM11253) was able to impair the effect of ATRA treatment in cancer stem cell population (Fig. 2i,j). On the other hand, RARβ antagonist (LE135) induced a potentiation of ATRA effect treatment on cancer stem cell self-renewal (Fig. 2i,j) indicating that RARβ might function as a buffer for retinoic acid response on cancer stem cell population.

### ATRA and Gö6976 combination modulates cell cycle progression, induces apoptosis and impairs autophagy

**Figure 3:**
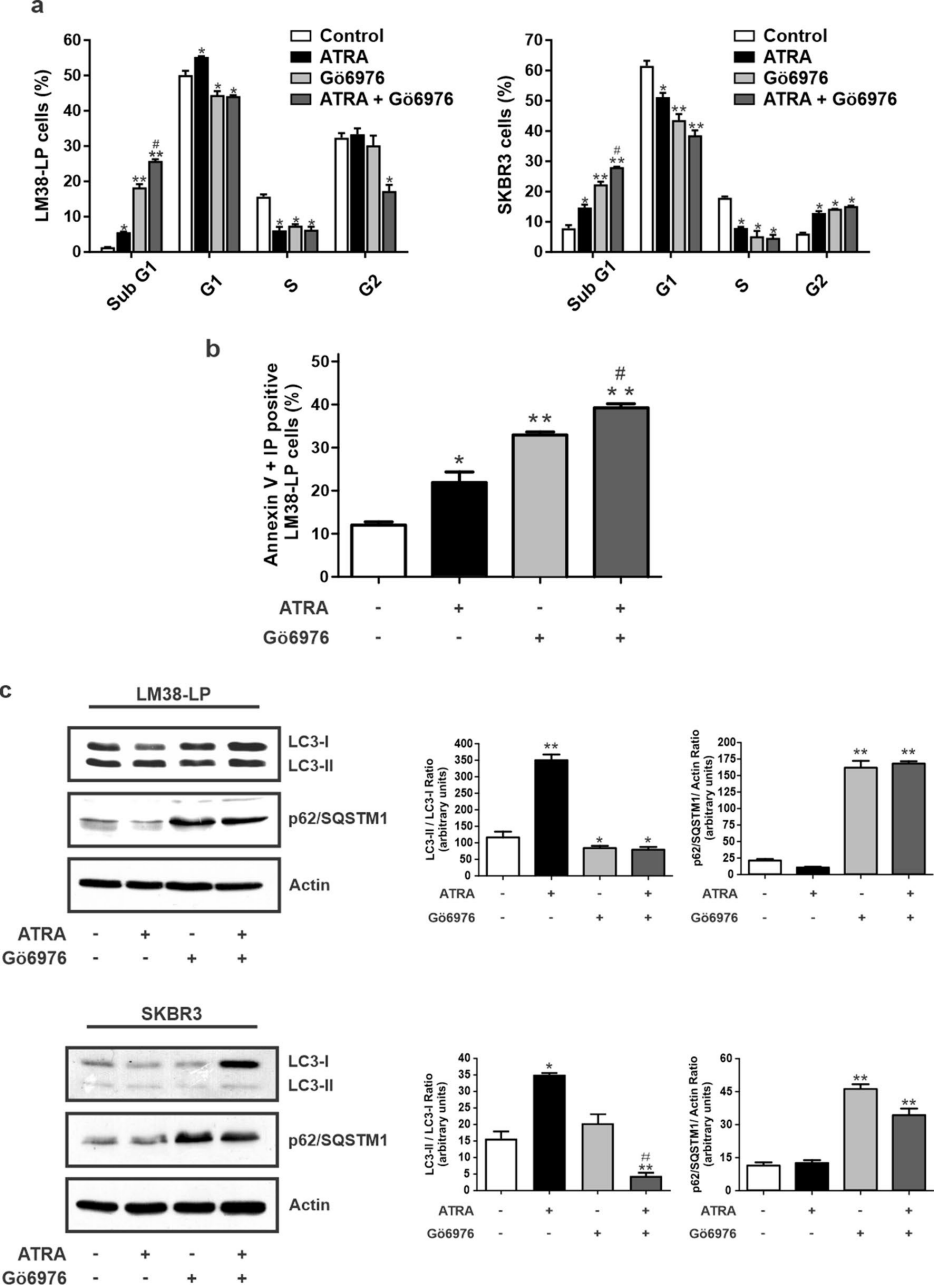
Modulation of cell cycle progression, apoptosis and autophagy. (**a**) Analysis of LM38-LP and SKBR3 cell cycle by flow cytometry after treatments with ATRA (0.5 µM) and/or Gö6976 (0.5 µM) or vehicle as control for 96 h. Histograms represent the mean ± S.D., *p < 0.05 versus control, **p < 0.01 versus control, ^#^*p* < 0.05 versus Gö6976 and ATRA (ANOVA test). Three independent experiments were performed. (**b**) Quantification of Annexin V staining by flow cytometry. LM38-LP cells were treated with ATRA (0.5 µM) and/or Gö6976 (0.5 µM) or vehicle as control for 96 h. Data represent the mean ± S.D, *p < 0.05 versus control, **p < 0.01 versus control, ^#^*p* < 0.05 versus Gö6976 and ATRA (ANOVA test). Three independent experiments were performed. (**c**) Immunoblot analysis and quantification of LC3 II/LC3 I and p62/SQSTM1 for LM38-LP and SKBR3 cells pre-treated with ATRA (0.5 µM) and/or Gö6976 (0.5 µM) or vehicle as control during 48 h. Results are representative of 3 independent experiments. Histograms represent mean ± S.D. of triplicate determinations, *p < 0.05 versus control, **p < 0.01 versus control, ^#^*p* < 0.05 versus Gö6976 and ATRA (ANOVA test).

Cell cycle distribution of LM38-LP and SKBR3 cells was analyzed after treatments with single agents or their combination. As compared to control cells, ATRA led to an increased accumulation of LM38-LP cells in the G_0_/G_1_ phase coupled with a reduction of the S phase and an increase of the sub G_1_ phase of cell cycle (Fig. 3a, left panel). In SKBR3 cells, ATRA treatment increased cells accumulation in G_2_ and sub-G_1_ phases in cell cycle, coupled with a reduction of cells in S and G_1_ (Fig. 3a, right panel). On the other hand, Gö6976 treatment induced a significantly increase in the Sub-G_1_ fraction on both cell lines (Fig. 3a). Although ATRA induced a slight but significant increase of this fraction, combined treatment showed a greater effect in accumulation of cells in the sub-G_1_ phase (Fig. 3a), suggesting apoptosis events. To confirm apoptosis induction, LM38-LP cells were collected and annexin V staining was evaluated after ATRA and/or Gö6976 treatments. Combined treatment showed a greater effect on apoptosis induction as shown in Fig. 3b.

Next, we wanted to elucidate whether Gö6976 could behave as an autophagy inhibitor, due the role of autophagy in the inhibition of the apoptosis process^34^. Through Western blot we analyzed LC3-II/LC3-I expression ratio and p62/SQSTM accumulation as a marker of autophagy process. We could determine that ATRA treatment significantly increased LC3-II/LC3-I ratio in both cell lines, without significant modulation in p62/SQSTM, which is compatible with an autophagy activation profile (Fig. 3c). On the other hand, Gö6976 treatment and its combination with ATRA led to a significant decrease of LC3-II/LC3-I protein ratio with a significant increase of p62/SQSTM1expression in both cell lines. Altogether these protein expression profile correlates with autophagy inhibition.

### ATRA and Gö6976 treatments impair migratory potential and soluble MMPs activity

**Figure 4:**
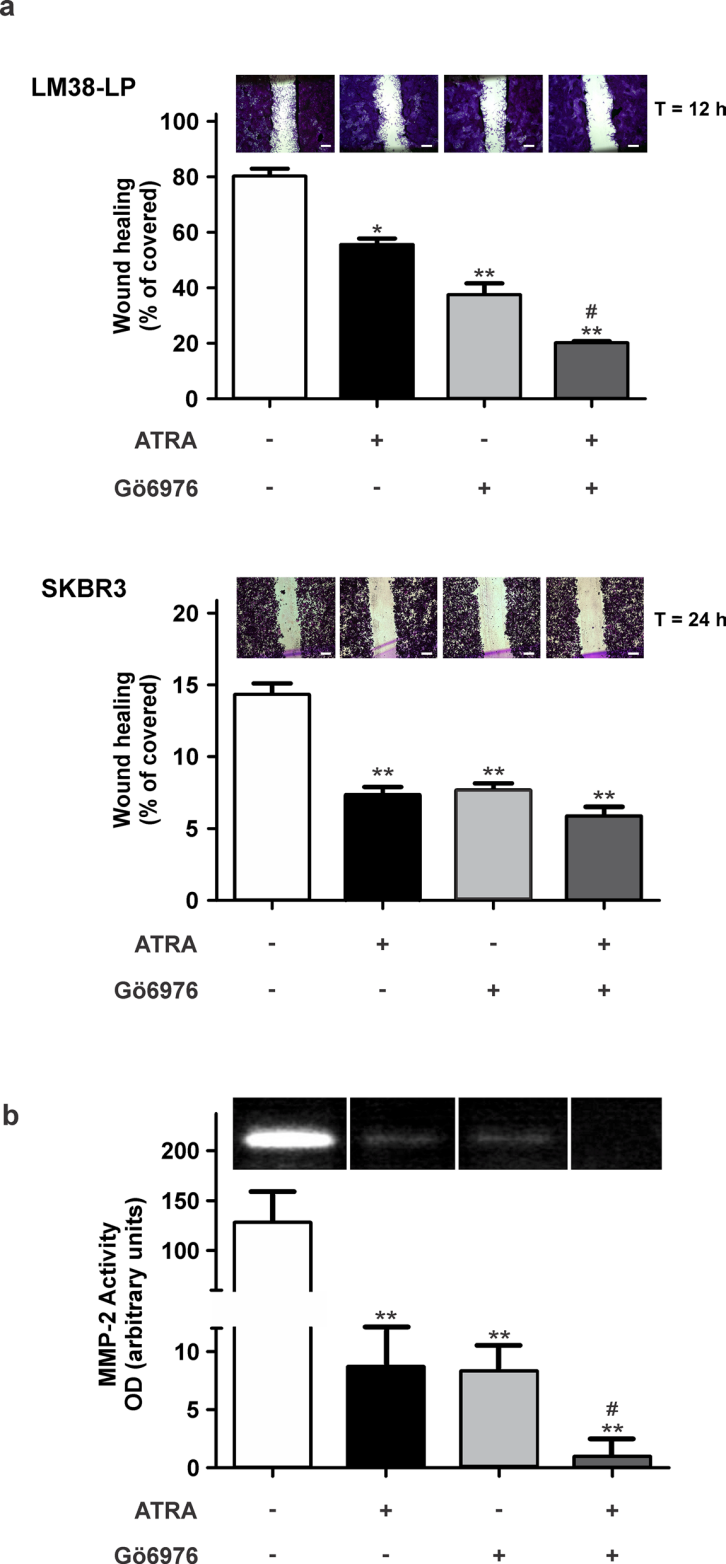
Modulation of migratory potential and soluble MMP-2 activity. (**a**) Monolayers pre-treated for 48 h with ATRA (0.5 µM) and/or Gö6976 (0.5 µM) or vehicle alone as control were “wounded” at time 0 and cells were allowed to migrate into the cell-free area for 12 h (LM38-LP cells) or 24 h (SKBR3 cells). Cell migration was quantified by calculating the percentage of area occupied by cells that migrated into the original cell-free wounded area. Data are expressed as mean ± S.D. of triplicate determinations, *p < 0.05 versus control, **p < 0.01 versus control, ^#^*p* < 0.05 versus Gö6976 and ATRA (ANOVA test). Three independent experiments were performed. Inset: Representative photographs of wounded monolayers at final time are shown (Scale bar = 50 μm)**:** Quantification of MMP-2 secreted activity of pre-treated LM38-LP monolayers. MMP-2 lytic bands were digitalized with a Photo/Analyst Express System and signal intensity was quantified with Gel-Pro Analyzer software. Data represent mean ± S.D. of triplicate determinations, **p < 0.01 versus control, ^#^*p* < 0.05 versus Gö6976 and ATRA (ANOVA test). At least 3 independent experiments were performed with similar results. Inset: Cropped bands corresponding to a representative zymogram are shown.

The effect of ATRA and Gö6976 combination on migratory potential of both LM38-LP and SKBR3 cells were analyzed through a “wound healing” assay as described in “Materials and Methods” section. Both ATRA and Gö6976 treatments significantly impaired LM38-LP cell migration towards the wounded area as compared to control cells. Moreover, combined treatment enhanced this inhibition (Fig. 4a, upper panel). SKBR3 cells have a very low migratory capacity, nevertheless, both treatments decreased migratory potential (Fig. 4a, lower panel). Additionally, we had explored soluble MMPs activity in LM38-LP cells since these proteases are intimately related to migratory and invasive processes. Both ATRA and Gö6976 treatments decreased secreted MMP-2 activity, being undetectable under combined treatment (Fig. 4b).

### ATRA and Gö6976 treatments modulate RARs expression

**Figure 5:**
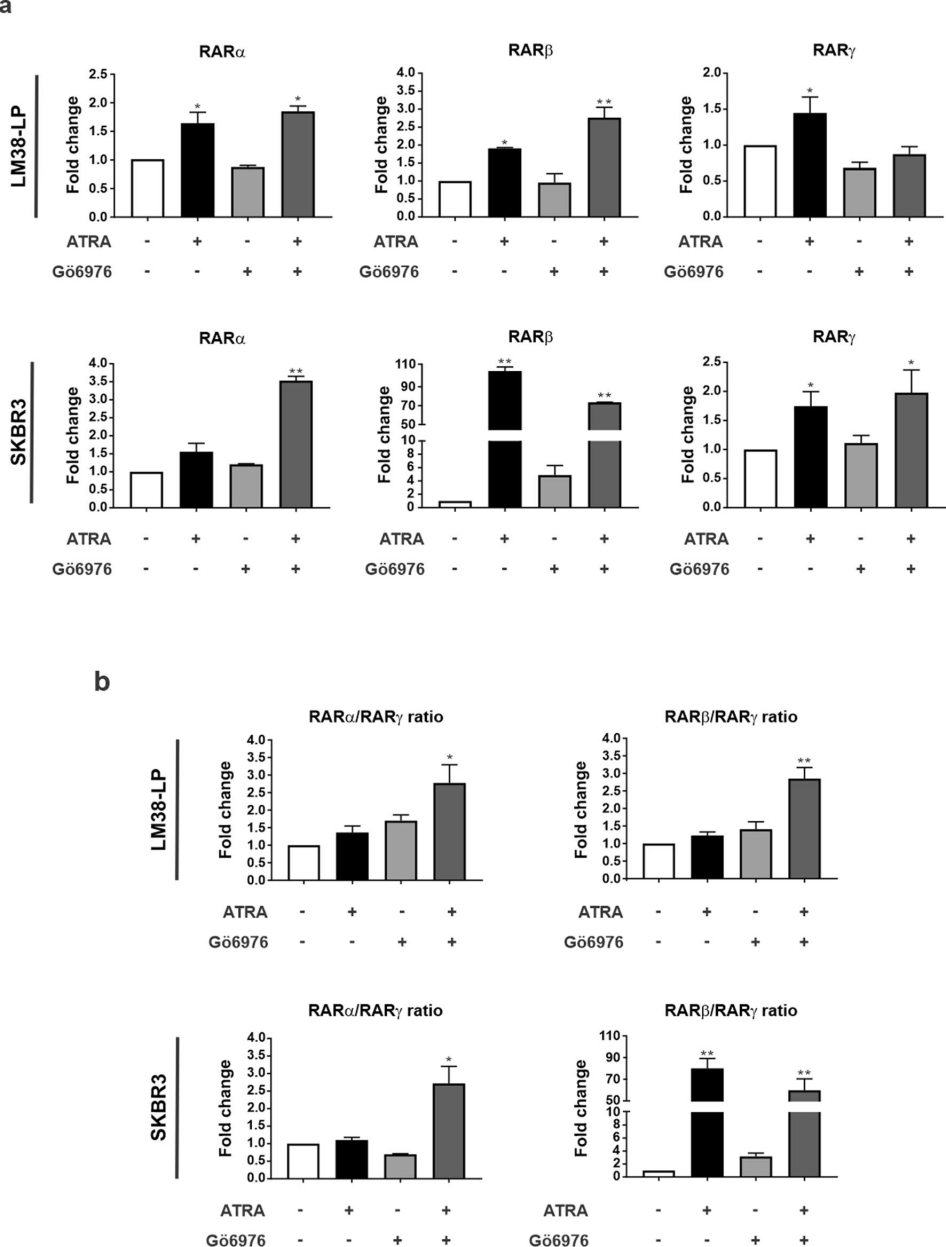
Modulation of Retinoic Acid Receptors. (**a**) LM38-LP monolayers were treated with ATRA (0.5 µM) and/or Gö6976 (0.5 µM) or vehicle as control for 48 h and then RNA was isolated. RARα, RARβ and RARγ expression was analyzed by RT-qPCR. The fold of change of mRNA levels was calculated by normalizing the absolute levels of RARs mRNA, using the ΔΔCt method with GAPDH used as an internal control. Histograms represent mean ± S.D., *p < 0.05 versus control, **p < 0.01 versus control (ANOVA test). Results are representative of three experiments. (**b**)Evaluation of RARα/RARγ and RARβ/RARγ expression ratio. LM38-LP and SKBR3 cells were treated with ATRA (0.5 µM) and/or Gö6976 (0.5 µM) or vehicle as control for 48 h. GAPDH was used as an internal control. Histograms represents mean ± S.D., *p < 0.05 versus control, **p < 0.01 versus control (ANOVA test).

By RT-qPCR we could determine that Gö6976 treatment alone did not alter RARs expression (Fig. 5a). On the other hand, ATRA induced a significant increase in RARβ and RARγ in both cell lines (Fig. 5a). When the combined condition was analyzed, both RARα and RARβ significantly increased their expression, while RARγ levels were affected only in SKBR3 cells (Fig. 5a). Given that RARs ratio is essential to elucidate the final cell’s fate in response to a treatment^35^, we analyzed RARα/RARγ and RARβ/RARγ ratio after ATRA and Gö6976 treatments. The combined treatment induced a significant increase of RARα/RARγ and RARβ/RARγ ratio in both cell lines, which is compatible with a differentiated cell profile (Fig. 5b).

### ATRA and Gö6976 treatment impaired in vivo tumor growth, metastatic dissemination and CSC frequency

**Figure 6:**
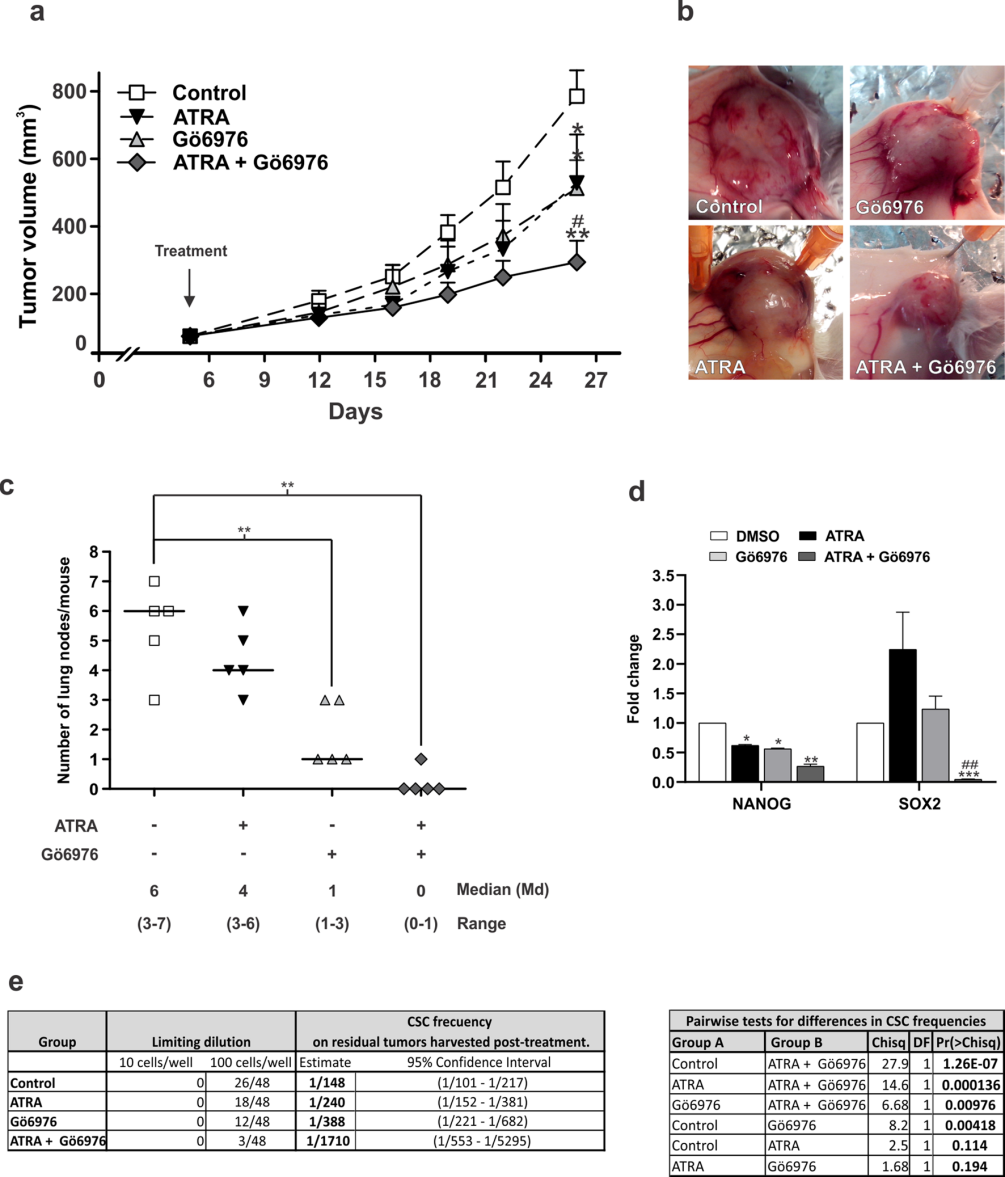
Evaluation of tumor growth, metastatic dissemination and cancer stem cell frequency. (**a**) LM38-LP cells were harvested from subconfluent monolayers and orthotopically inoculated into the fat pad of BALB/c mice. Five days later, animals receive the different treatments that consist in a silastic pellet containing ATRA (10 mg) or an empty pellet as control. Mice additionally received a peritumoral injection of Gö6976 (0.2 mg/kg) in physiological solution or physiological solution as control. Size of the two perpendicular diameters was recorded and used to calculate tumor volume. Each data point represents mean ± S.D. (n = 5), *p < 0.05 versus control, **p < 0.01 versus control, ^#^*p* < 0.05 versus Gö6976 (ANOVA test). Two independent experiments were performed with similar results. (**b**) Representative photographs of LM38-LP tumors at necropsy are shown. (**c**) The number and size of surface lung nodules was determined under a dissecting microscope. Each data point represents the number of lung nodules per animal. Median and range are indicated in each experimental group. **p < 0.01 versus control (Kruskall-Wallis test). Figure shows the results of one experiment representative of three independent assays. (**d**) RNA from LM38-LP tumors harvested post-treatment was isolated. Nanog and Sox2 expression was analyzed by RT-qPCR. The fold of change of mRNA levels was calculated using the ΔΔCt method with GAPDH used as an internal control. Histograms represent mean ± S.D. *p < 0.05 versus control, **p < 0.01 versus control, ***p < 0.001 versus control, ^##^*p* < 0.01 versus Gö6976 (ANOVA test). Three independent experiments were performed. (**e**) CSC frequency estimates and *p* values calculated by using the ELDA software are shown.

Next, we evaluate whether in vitro described results had an in vivo correlation. Twenty female BALB/c mice were orthotopically inoculated with LM38-LP cells and five days later, animals received treatments as described in “Materials and Methods” section (5 animals per group, assays were performed twice and all animals 20/20 presented good health status). We could determine that both ATRA and Gö6976 treatments reduced LM38-LP in vivo tumor growth (Fig. 6a,b). Once again, ATRA/Gö6976 combined significantly impairs in vivo tumor growth when compared with each treatment alone. (Fig. 6a,b). Although Gö6976 treatment alone induced a reduction in lung metastatic capacity, it is important to note that, in the combined treatment group, just one of tumor bearing mice developed one metastatic focus (Fig. 6c). Furthermore, only combined treatment was able to significantly reduce both NANOG and SOX2 expression (Fig. 6d). Finally, an ELDA was performed using 10 and 100 cells derived from LM38-LP tumors harvested post-treatment, in order to evaluate CSC frequency. ELDA results revealed that ATRA/Gö6976 combined treatment led to a significantly lower CSC frequency as compared to the vehicle (1/1710, *p* = 1.26E-07), as well as to ATRA (1/240, *p* = 0.000136) or Gö6976 (1/388, *p* = 0.00976) treatment alone (Fig. 6e).

### High RARα mRNA expression and low PKCα mRNA expression predicts favorable survival of estrogen receptor negative breast cancer patients

Finally, we decided to perform a bioinformatic analysis from Kaplan Meier Plot database^31^ to analyze whether Protein Kinase C Alfa (PRKCA) and/or Retinoic Acid Receptor Alfa (RARA) mRNA expression level correlates with relapse free survival (RFS) in ER negative breast cancer patients (same condition displayed by the employed cell lines).

**Figure 7:**
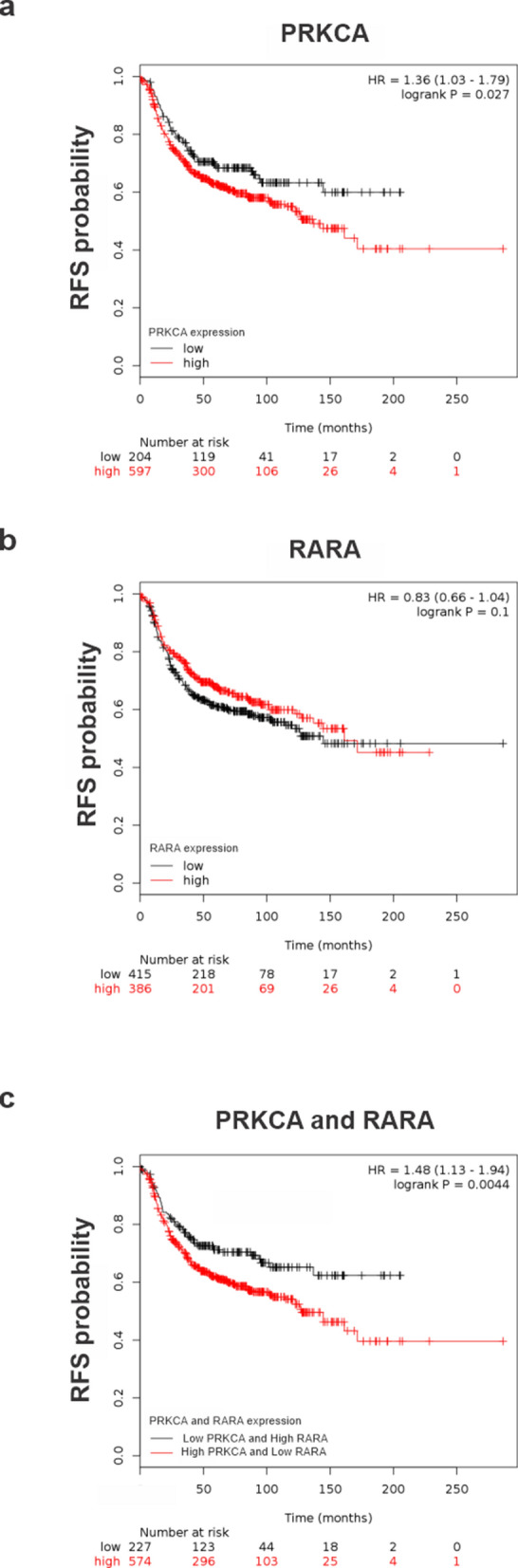
Bioinformatic analysis of PKCα (PRKCA) and RARα (RARA) mRNA expression. (**a**) Kaplan–Meier plots for PRKCA in negative estrogen receptor breast cancer cohorts. (**b**) Kaplan–Meier plots for RARA in negative estrogen receptor breast cancer cohorts. (**c**) Kaplan–Meier plots for the mean expression of RARA and PRKCA in negative estrogen receptor breast cancer cohorts. Log-rank p values and hazard ratios (HRs; 95% confidence interval in parentheses) are shown.

Low PRKCA mRNA expression was statistically associated with a best RFS of breast cancer patients with negative estrogen receptor tumors (HR = 1.36, *p* = 0.027) (Fig. 7a), while RARA mRNA expression did not affect RFS probability (Fig. 7b). Then, we analyzed the impact of PRKCA mRNA and RARA mRNA expression on RFS of estrogen receptor negative breast cancer patients. Interestingly, low PRKCA mRNA expression together with high RARA mRNA expression showed greater RFS (HR = 1.48, *p* = 0.0044) than low mRNA PRKCA levels alone (Fig. 7c). These results reinforce the importance of proposed ATRA and Gö6976 combined treatment for estrogen receptor negative breast cancer patients.

## Discussion

Among women, breast cancer is the most frequently diagnosed cancer in the vast majority of countries around the world and is also the leading cause of cancer death in over 100 countries^1^. Hormone-independent breast cancers are considered a high-risk group since patients present an unfavorable prognosis with high recurrence rates^36^. Targeted therapy has changed the course of breast cancer treatment, but blocking a single pathway is finally ineffective, due to the activation of redundant and/or alternative oncogenic pathways^37^. Therefore, it is imperative to develop new therapies, targeting different signaling pathways, in order to generate a great impact on the evolution of this disease.

ATRA, as well as its natural and synthetic derivatives collectively known as retinoids, are promising agents for treatment or chemoprevention of different malignancies including breast cancer. Although in clinical settings, the use of retinoic acid as monotherapy has been controversial for solid tumors, some phase II clinical trials are still being evaluated^38,39^. However, several authors have demonstrated the effectiveness of retinoid therapy in combination with other drugs such as tamoxifen or trastuzumab^40^, suggesting that retinoic acid utility depends on its capacity to potentiate the effect of other compounds employed for cancer treatment^15,39,41^.

The importance of performing a combined treatment relies in the fact that ATRA can activate other kinases through a non-canonical pathway. In fact, several studies have reported that retinoids can activate some PKC isoforms^42–45^. So, our rationale was to potentiate the differentiator effect of ATRA by combining this retinoid with a classical PKC inhibitor thus avoiding ATRA undesired effects.

As shown in results section, ATRA and Gö6976 combined treatment highly reduced proliferative capacity of breast cancer cell lines in a synergistic manner. To elucidate mechanisms underlying this proliferation inhibition, we examined the effect of both single agents and their combination on autophagy, since it has been described that this mechanism impairs apoptosis^46^. Although, combined treatment induced a significant apoptosis increase when compared with each treatment alone, it has been reported that ATRA induced autophagy in hormone-independent breast cancer cell lines employed^47^. Nevertheless, apoptosis induction caused by Gö6976 treatment prevailed over ATRA effect, demonstrating that blocking autophagy could be an interesting strategy to potentiate ATRA effect on apoptosis.

Regarding cancer stem cell population, we could observe that primary mammospheres pre-treatment with ATRA, favors the preservation of stem/progenitor cells with the potential to regenerate the original cell line. This was reflected both as an increase in secondary mammospheres number, as well as in the clonogenic capacity. However, secondary mammospheres obtained after ATRA treatment showed a reduction in growth rate, evidenced by the decrease in their diameter. In sum, while an increase in the self-renewal capacity of CSC could be detected, these cells display a lower growth rate.

To evaluate the involvement of retinoid receptors in these results, we employ specific RARβ and RARγ antagonists^15^. We observed that RARγ activation by ATRA was involved in CSC population growth. These results correlate with previous publications, where RARγ is described as a key receptor involved in hematopoietic cells self-renewal^48^. On the other hand, ATRA treatment also increases RARβ levels showing that retinoic acid receptors system is activated and functional. It is important to note that RARβ promoter contains RARE sequences, allowing this gene transcription after retinoid stimulus. Due to its role in cell differentiation, pharmacological inhibition of RARβ activity increased even more CSC self-renewal.

Regarding Gö6976 effects, we observed that the treatment with this PKC inhibitor blocks both self-renewal and clonogenic capacity of CSC, confirming that PKCα is a critical signaling component for CSC^18^, also impairing the negative effects exerted by ATRA over that cell population.

Several in vitro features associated with tumor progression were also analyzed. In this sense, ATRA/Gö6976 combined treatment reduced migratory capacity and MMP-2 secreted activity better than each treatment alone. Consistently, in vivo metastatic capability was also affected. In fact, combined treatment not only affected the metastatic potential but also produced an important impairment of in vivo tumor growth.

It is well known that RARα is one of the key members in the response to ATRA treatment. Acute promyelocytic leukemia patients have a malfunction of RARα protein due to a genetic fusion between RARα and PML kinase^49^. This genetic abnormality causes a lack of response to basal plasma levels of retinoic acid, leading to undifferentiated immune cells^50^. In these malignancies, increasing Retinoic Acid levels in plasma led to the differentiation of immune cell, allowing total cure of this disease. For this reason, we focus on how combined treatment modulates RARs expression. Only combined treatment led to a significant increase of RARα levels, probably indicating the induction of a differentiated phenotype and therefore reducing malignant potential. Regarding RARβ, ATRA or the combined treatment, increased its expression. This receptor acts as a tumor suppressor^51^, and generally is downregulated or not expressed in breast cancers^52^. RARγ display a proliferative role in hepatocellular carcinoma^53^ and in breast cancer^35^. At the same time this receptor is involved in hematopoietic stem cell self-renewal^48^. Our studies reveal an upregulation of RARγ induced by ATRA treatment. Nevertheless, the combination with Gö6976 impaired induction of this retinoid receptor in LM38-LP cells. Furthermore, it has been described that RARα/RARγ and RARβ/RARγ expression ratio is critical to elucidate the response to ATRA treatment^35^. Only combined treatment increased both ratios, leading to a response compatible with the reversion of the malignant phenotype driving to a differentiated state^35^.

Finally, it is important to note that the cell lines used in the present study express PKCα. It has been reported that this PKC isoform could be considered as a poor prognosis marker in breast cancer^17^, thus we decided to perform an *in-silico* analysis in order to evaluate how PKCα and RARα mRNA expression levels affected the RFS probability in hormone receptor negative breast cancer patients. Our analysis showed that low PRKCA mRNA expression together with high RARA mRNA expression becomes a favorable factor of prognosis for these patients.

In sum, our findings show that Gö6976 treatment potentiates antitumor effect of ATRA by inducing apoptosis of breast cancer cells, inhibiting cancer stem cell self-renewal and clonogenicity and leading to RARs balance compatible with an anti-oncogenic response. Moreover, in vivo tumor growth, metastasis spreading, and CSC frequency were also inhibited by the combined treatment in the LM38-LP triple negative mammary cancer cell line.

The importance of our findings relies in the fact that experimental models employed are hormone-independent tumors. Although clinical treatment of these pathologies is chemo and radiotherapy, these malignancies present high recurrence rate and/or acquire treatment-resistance and to date, there is no effective or directed therapy. For this reason, the development of pre-clinical assays imperative in order to propose novel therapies, such as the presented in this work.

The rationale design of molecules that block classical PKCs activity, in combination with retinoids could led the development of new potential therapies for the treatment of hormone-independent breast cancer patients.

## Supplementary Information

Supplementary Information.

## Data Availability

All the data generated during this study are included in this article.
