# Supplementary material for: All-trans retinoic acid and protein kinase C α/β1 inhibitor combined treatment targets cancer stem cells and impairs breast tumor progression
Source: Sci Rep. 2021 Mar 15;11:6044. doi: 10.1038/s41598-021-85344-w (PMC7961031; doi:10.1038/s41598-021-85344-w)

**All-Trans Retinoic Acid and Protein Kinase C** **α/β1 inhibitor combined treatment targets cancer stem cells and impair breast tumor progression**

**Damian Emilio Berardi^1,2^, Lizeth Ariza Bareño^1^, NataliaAmigo^1^, Luciana Cañonero^3^, Maria de las Nieves Pelagatti^1^, Andrea Nora Motter^4^, María Agustina Taruselli^1^, María Inés Díaz Bessone^1,6^, Stefano Martin Cirigliano^1,5^, Alexis Edelstein^4^, María Giselle Peters^1,7^, Miriam Diament^1^, Alejandro Jorge Urtreger^1,7^ and Laura Beatriz Todaro^1,7^.**

^1^ Universidad de Buenos Aires, Instituto de Oncología Ángel H. Roffo, Área Investigación, Buenos Aires, Argentina.

^2.^Current address: The Ben May Department for Cancer Research, The Gordon Center for Integrative Sciences, The University of Chicago, Chicago, IL, USA.

^3^ Universidad de Buenos Aires, Facultad de Ciencias Exactas y Naturales, Departamento Química Biológica, CONICET- Universidad de Buenos Aires, Instituto de Química Biológica de la Facultad de Ciencias Exactas y Naturales (IQUIBICEN), Buenos Aires, Argentina

^4^ Unidad Operativa Centro de Contención Biológica de la Administración Nacional de Laboratorios e Institutos de Salud (UOCCB-ANLIS) “Dr. Carlos G. Malbrán”, Buenos Aires, Argentina.

^5^ Current address: Meyer Cancer Center, Weill Cornell Medicine, New York, NY, USA.

^6^ Current address: Universidad Nacional de San Martín, Instituto de Nanosistemas, Campus Miguelete, San Martín, Argentina.

^7^Member of the Scientific Research Career of the Consejo Nacional de Investigaciones Científicas y Técnicas (CONICET), Buenos Aires, Argentina.

**Contact information:**

- Damian Emilio Berardi E-mail: [damianberardi@gmail.com](mailto:damianberardi@gmail.com)
- Lizeth Ariza Bareño E-mail: [LISA19111@hotmail.com](mailto:LISA19111@hotmail.com)
- Natalia Amigo E-mail: [nlamigo82@gmail.com](mailto:nlamigo82@gmail.com)
- Luciana Cañonero E-mail: [lucianacanonero@gmail.com](mailto:lucianacanonero@gmail.com)
- Maria de las Nieves Pelagatti E-mail: [marinipelagatti@yahoo.com.ar](mailto:marinipelagatti@yahoo.com.ar)
- Andrea Nora Motter E-mail: [andreamotter@gmail.com](mailto:andreamotter@gmail.com)
- María Agustina Taruselli E-mail: [ma.taruselli@gmail.com](mailto:ma.taruselli@gmail.com)
- María Inés Díaz Bessone E-mail: [ine.db84@gmail.com](mailto:ine.db84@gmail.com)
- Stefano Martin Cirigliano E-mail: [smcirigliano@gmail.com](mailto:smcirigliano@gmail.com)
- Alexis Edelstein E-mail: [Aedel@anlis.gov.ar](mailto:Aedel@anlis.gov.ar)
- María Giselle Peters E-mail: [mpeters@fmed.uba.ar](mailto:mpeters@fmed.uba.ar)
- Miriam Diament E-mail: [miriam.diament@gmail.com](mailto:miriam.diament@gmail.com)
- Alejandro Jorge Urtreger E-mail: [urtreger@yahoo.com.ar](mailto:urtreger@yahoo.com.ar)
- *Laura Beatriz Todaro E-mail: [ltodaro@gmail.com](mailto:ltodaro@gmail.com) / [ltodaro@institutoroffo.uba.ar](mailto:ltodaro@institutoroffo.uba.ar)

**SUPPLEMEMENTARY FIGURE**

**
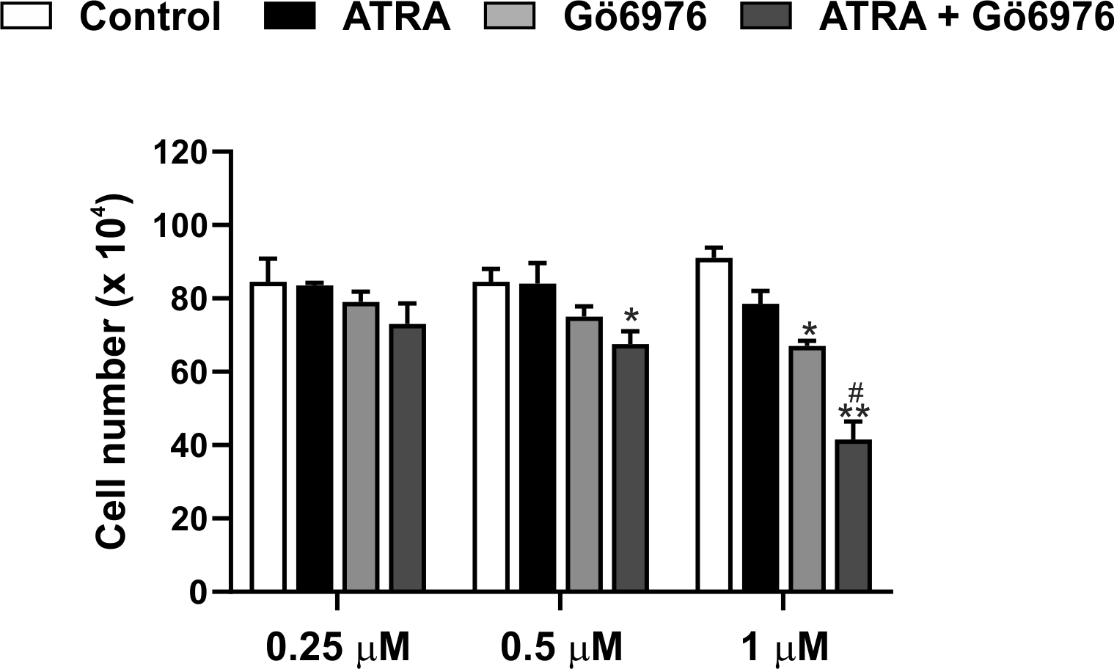
**

**SUPPLEMENTARY FIGURE LEGEND**

**Supplementary Figure S1.** HCC38 cell number was assessed 96 h after treatments with ATRA (0.25-1 µM) and/or Gö6976 (0.25-1 µM) or vehicle as control. Data represent the mean ± S.D. *p<0.05 *vs*. control, **p<0.01 *vs*. control, ^#^p<0.05 *vs*. Gö6976, (ANOVA test). Three independent experiments were performed

**SUPPLEMEMENTARY TABLE**

**Table S1.** List of primers used for quantitative real time PCR.

| **Primer**  **(RT-qPCR)** | **Forward** | **Reverse** |
| --- | --- | --- |
| Human RARα | GGTCGGCGATGGTGAGGGT | TGGGCAAATACACTACGAACAACAG |
| Human RARβ | GATTTCTACACTGCGAGTCCGTC | GTGGAGATGGGGGGCTTG |
| Human RARγ | TCAAAGCTGCCTGCCTAGAT | GTCGGAGAAGGTCATGGTGT |
| Mouse RARα | GGCAAGTACACTACGAACAA | GCGAACTCCACAGTCTTAAT |
| Mouse RARβ | GTACGGCTCTGTTCTTTCTATAC | GTGGATGTACAGTGGTTTACTC |
| Mouse RARγ | TTCTAACTGGCTCCCTCTAC | GAAAGGCAGTGCTGAGATT |
| Human Nanog | ACCTTGGCTGCCGTCTCTGG | AGCAAAGCCTCCCAATCCCAAACA |
| Human Sox2 | GAGCTTTGCAGGAAGTTTGC | GCAAGAAGCCTCTCCTTGAA |
| Mouse Nanog | CACAGTTTGCCTAGTTCTGAGG | GCAAGAATAGTTCTCGGGATGAA |
| Mouse Sox2 | GCGGAGTGGAAACTTTTGTCC | GGGAAGCGTGTACTTATCCTTCT |
| Human/Mouse GAPDH | TGCACCACCAACTGCTTAGC | GGCATGGACTGTGGTCATGAG |


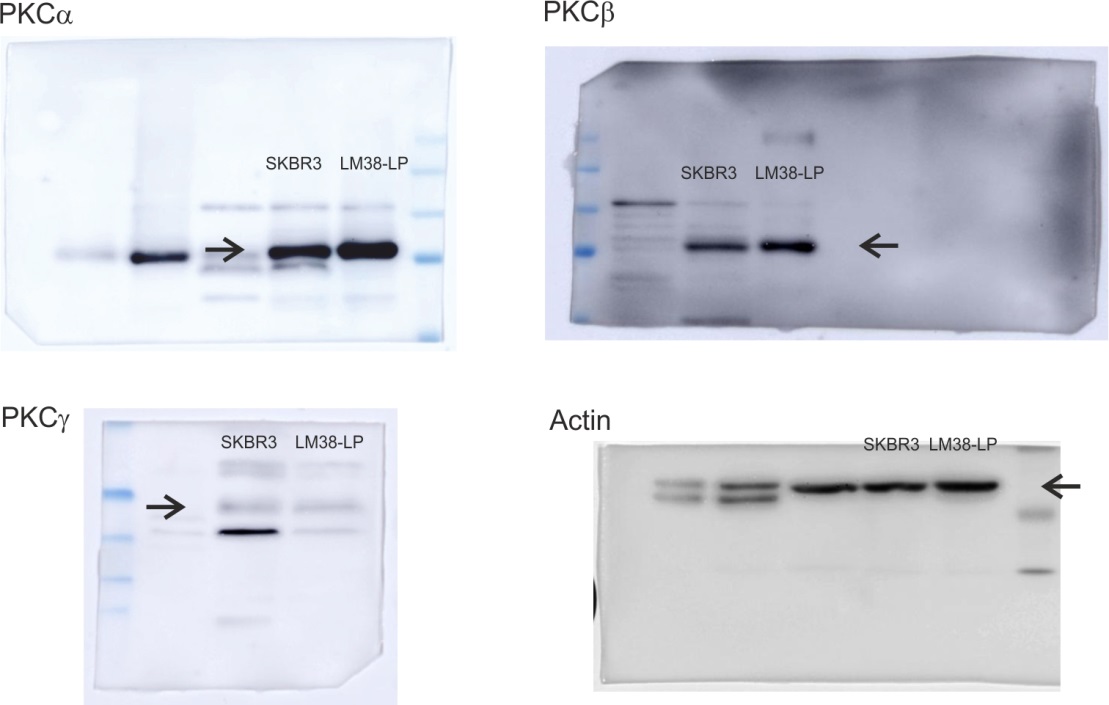
Figure 1a- Western Blots

Figure 3c- Western blots


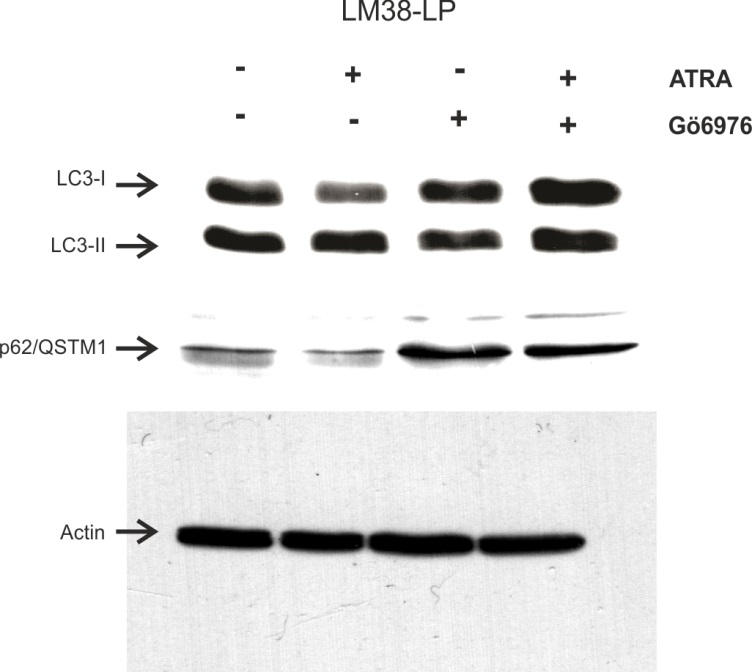

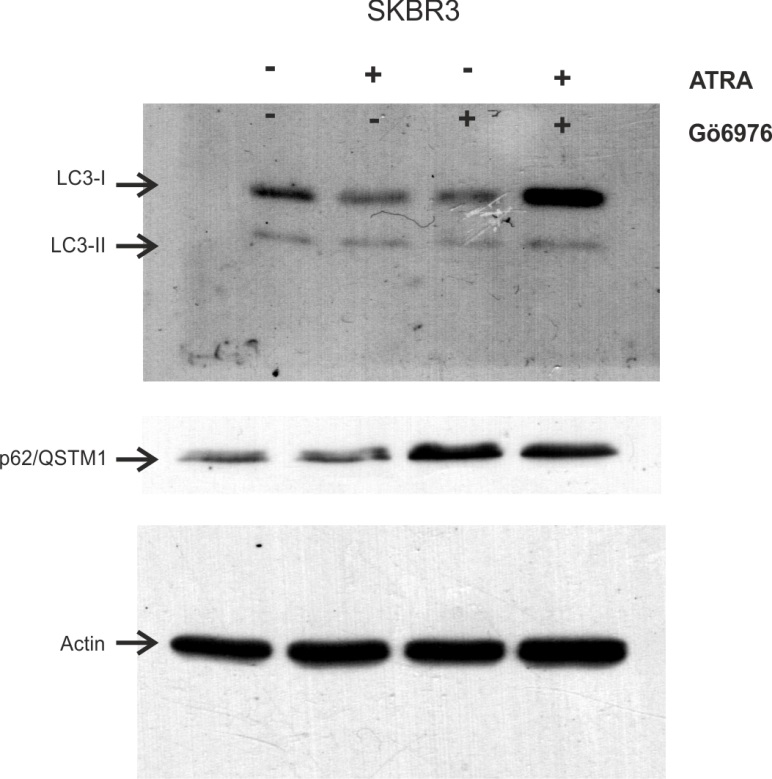


Figure 4b inset - zymography


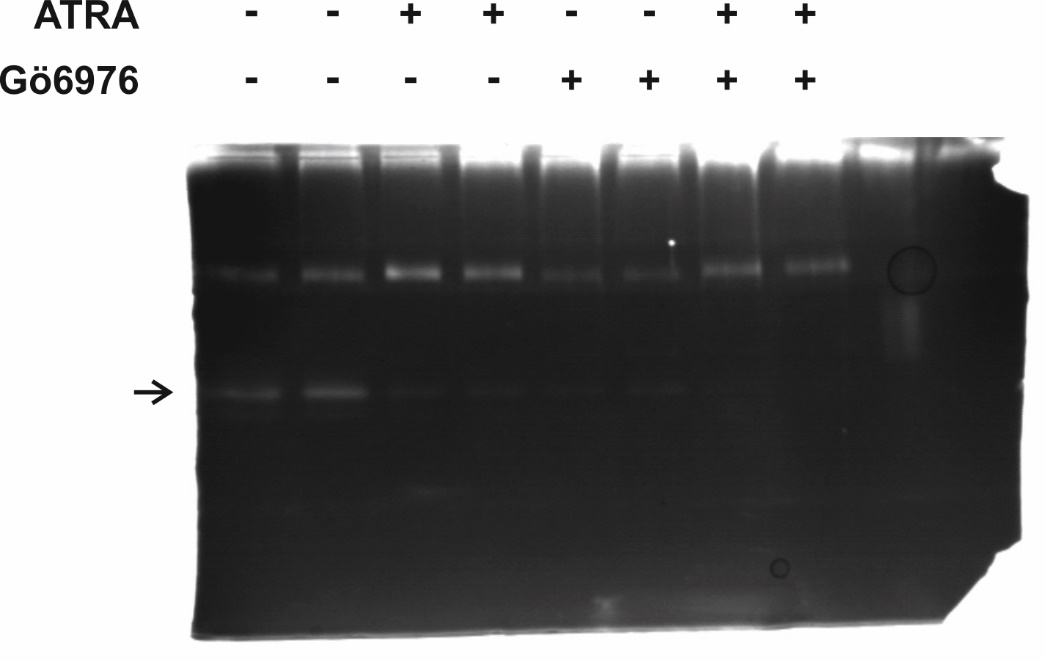

Supplement: Supplementary file 1 — Supplementary Information. [file 41598_2021_85344_MOESM1_ESM.docx]
